# Real-world serplulimab plus chemotherapy for extensive-stage small cell lung cancer in china: an external validation of ASTRUM-005 trial

**DOI:** 10.1186/s12916-026-05055-9

**Published:** 2026-07-15

**Authors:** Lin Wu, Chengping Hu, Wenzhong Su, Hui Zhu, Wei Hong, Haichuan Su, Yan Yu, Tao Luo, Zhigang Liu, Zhentian Liu, Qing Pu, Caixia Wang, Xuhong Min, Hui Guo, Yifei Chen

**Affiliations:** 1 The Department of Thoracic Medical Oncology, Hunan Cancer Hospital/The Affiliated Cancer Hospital of Xiangya School of Medicine, Central South University, Changsha, 410031 Hunan Province China; 2 Department of Respiratory Medicine, Xiangya Hospital, Central South University, Changsha, 410008 Hunan Province China; 3 Department of Respiratory Medicine, Shanxi Cancer Hospital, Taiyuan, 030000 China; 4 Department of Radiation Oncology, Shandong Cancer Hospital and Institute, Shandong First Medical University and Shandong Academy of Medical Science, Jinan, 250117 China; 5 Department of Chest Oncology, Zhejiang Cancer Hospital, Hangzhou, 310022 China; 6 Department of Oncology, Tangdu Hospital, Air Force Medical University, Xi’an, 710038 China; 7 Harbin Medical University Cancer Hospital, Harbin, 150081 China; 8 Department of Respiratory and Critical Care Medicine, The Affiliated Hospital of Xuzhou Medical University, Xuzhou, 221004 China; 9 Cancer Center, Guangdong Engineering Research Center of Boron Neutron Therapy and Application in Malignant Tumors, Dongguan Key Laboratory of Precision Diagnosis and Treatment for Tumors, The Tenth Affiliated Hospital, Southern Medical University (Dongguan People’s Hospital), Dongguan, 523059 China; 10 Department of Thoracic Medical Oncology, Jiangxi Cancer Hospital, Nanchang, 330001 China; 11 Department of Medical Oncology, The First Affiliated Hospital of Guangxi Medical University, Nanning, 530022 China; 12 Chemotherapy Department of Oncology Center, Shandong Provincial Hospital Affiliated to Shandong First Medical University, Jinan, 250021 China; 13 Department of Oncology Radiotherapy, Anhui Chest Hospital, Hefei, 230000 China; 14 Department of Medical Oncology, The Second Affiliated Hospital of Xi’an, Jiaotong University, Xi’an, 710004 China

**Keywords:** Serplulimab, Extensive-stage small cell lung cancer (ES-SCLC), Real-world evidence (RWE), Chemoimmunotherapy, Immunotherapy maintenance

## Abstract

**Background:**

In the randomized phase 3 ASTRUM-005 trial, adding serplulimab to chemotherapy improved survival in extensive-stage small cell lung cancer (ES-SCLC), but whether these results generalize to routine practice remains uncertain. To evaluate the real-world effectiveness and safety of first-line serplulimab plus chemotherapy in China and to benchmark outcomes against the pivotal trial using a matching-adjusted indirect comparison (MAIC).

**Methods:**

This nationwide, observational study enrolled ES-SCLC patients treated with serplulimab and investigator-selected chemotherapy across 14 sites in China. For subsequent analyses, patients were grouped by alignment with the ASTRUM-005 trial regimen: an RCT-regimen cohort and a real-world regimen cohort. The primary endpoints were overall survival (OS) and real-world progression-free survival (rwPFS); secondary endpoints included objective response rate (ORR) and safety. The MAIC weighted eligible real-world patients to the aggregate baseline profile of the pivotal trial.

**Results:**

Among 635 patients, the median OS was 17.20 months (95% CI, 15.43–19.77) and the median rwPFS was 8.17 months (95% CI, 7.40–9.20). The ORR was 69.05%, with comparable efficacy observed between RCT-regimen and real-world cohorts. Poor performance status and liver metastases independently predicted higher mortality. In the MAIC, OS in the RCT-regimen cohort was comparable to the serplulimab arm of the pivotal ASTRUM-005 trial (weighted log-rank *P* = 0.784). Safety was manageable and consistent with known profiles.

**Conclusions:**

This large-scale real-world study confirms the efficacy of serplulimab plus chemotherapy and tolerability in diverse ES-SCLC population, supporting the external validity and generalizability of the pivotal ASTRUM-005 trial results.

**Clinical trial registration:**

ClinicalTrials.gov (NCT06748495).

**Supplementary Information:**

The online version contains supplementary material available at https://doi.org/10.1186/s12916-026-05055-9.

## Background

Small-cell lung cancer (SCLC) accounts for approximately 15% of lung cancers and carries a dismal prognosis, reflecting its neuroendocrine biology and strong association with tobacco exposure [1]. More than 70% of patients presenting with extensive-stage SCLC at initial diagnosis, defined by distant spread beyond a tolerable radiation field. Over the past decade, several phase 3 trials have shown that adding immune checkpoint blockade to platinum-etoposide can extend survival in ES-SCLC. Among these, the anti-programmed cell death protein-1 (anti-PD-1) monoclonal antibody (mAb) serplulimab improved overall survival (OS) versus chemotherapy alone in the double-blind ASTRUM-005 trial (median, 15.8 vs. 11.1 months; hazard ratio [HR], 0.60; *P* < 0.001), and increased long-term survival (4-year OS, 21.9% vs. 7.2%) [2–5]. These data supported approval of serplulimab plus chemotherapy as a first-line standard of care in China [6], with growing uptake across Asia and Europe.

Despite these promising findings, clinical trial results may have limited generalizability to real-world populations due to inherently strict eligibility criteria. In ASTRUM-005, patients with Eastern Cooperative Oncology Group performance status (ECOG PS) ≥ 2, uncontrolled comorbidities, or untreated brain metastases were excluded, yet such features are frequently encountered in routine practice [7]. To date, no large-scale real-world study has systematically evaluated first-line serplulimab plus chemotherapy in ES-SCLC or benchmarked routine-care outcomes against ASTRUM-005 using rigorous comparative methods. Accordingly, we conducted a multicenter, observational study across 14 centers in China to evaluate the real-world effectiveness and safety.

## Methods

### Study design and patients

This ASTRUM-005R study (NCT06748495) was an investigator-initiated, registry-based, retrospective-prospective, nationwide observational trial across 14 Chinese cancer centers. The objectives were to evaluate the real-world effectiveness and safety of first-line serplulimab plus chemotherapy for ES-SCLC, and to assess the generalizability of pivotal ASTRUM-005 trial. Patient enrollment was initiated in April 2022 and completed in June 2024, with final data collection concluded in December 2024. Eligible adults (≥ 18 years) had pathologically confirmed ES-SCLC and initiated first-line serplulimab with investigator-selected chemotherapy. Inclusion required ≥ 2 cycles of serplulimab-based therapy and ≥ 1 post-baseline tumor assessment when measurable disease was present. Patients were excluded for missing essential clinical data, refusal of follow-up, or concurrent active malignancies. As an observational study, treatments followed routine practice per guidelines and institutional standards without protocol-mandated interventions. For subsequent analyses, patients were stratified by alignment with the landmark ASTRUM-005 regimen: the RCT-regimen cohort received serplulimab plus carboplatin-etoposide with four cycles and had baseline ECOG PS < 2; the real-world regimen cohort deviated from this protocol (e.g., > 4 combination cycles or use of alternative platinum agents).

The study was conducted in accordance with the ethical principles outlined in the Declaration of Helsinki and was reported following the Strengthening the Reporting of Observational Studies in Epidemiology (STROBE) guidelines (Additional file 1). The study protocol was approved by the Institutional Ethics Review Board of Hunan Cancer Hospital (approval number: 2024KS10), and all participating centers provided institutional approval. Written informed consent was obtained from all enrolled patients.

### Data collection and outcomes

Clinical data were collected prospectively or retrospectively from medical records. Baseline information included demographic, clinical, pathological, disease stage, and relevant laboratory and imaging data. Treatment details, including chemotherapy regimen, serplulimab exposure, radiotherapy, and follow-up, were systematically documented. Data were recorded in a centralized electronic data capture (EDC) system. Investigators at each center entered data, which was reviewed by the principal investigator (Lin Wu) for consistency. Discrepancies were addressed with site queries, and unresolved issues were adjudicated with another principal investigator (Chengping Hu).

Primary endpoints were OS and real-world progression-free survival (rwPFS). Secondary endpoints included objective response rate (ORR) and safety. An exploratory analysis used matching-adjusted indirect comparison (MAIC) to compare survival outcomes with ASTRUM-005. OS was defined from first-line serplulimab initiation to death from any cause. The rwPFS was defined as the time from treatment initiation to progression or death. Tumor response was assessed according to the Response Evaluation Criteria in Solid Tumors (RECIST), version 1.1. ORR was the proportion achieving complete or partial response (CR/PR). Treatment tolerability was evaluated based on the frequency, severity, and type of adverse events (AEs), graded by the Common Terminology Criteria for Adverse Events (CTCAE), version 5.0.

### Statistical analysis

All statistical analyses were conducted using R software (version 4.3.2).

#### Descriptive statistics and comparative assessments

Continuous data were shown as mean ± standard deviation (S.D.) if normally distributed, or as medians with interquartile ranges (IQRs) or 95% CIs when skewed. Categorical data were summarized as counts and proportions. The ORR and 95% CI were computed using the Clopper-Pearson method. Survival endpoints were estimated using the Kaplan-Meier approach, with median values with 95% CIs. Follow-up duration was determined using the reverse Kaplan-Meier method. Comparisons between subgroups for time-to-event outcomes used the log-rank test, and rate differences were examined with a chi-squared test or Fisher’s exact test. A landmark analysis of the RCT-regimen and real-world regimen cohorts at 2.8 months (corresponding to 4 treatment cycles) was performed to evaluate outcomes based on treatment duration and to minimize immortal time bias. Cox regression explored associations between baseline characteristics and rwPFS and OS. In addition, to assess the effect of maintenance therapy, time-dependent Cox regression models were applied, with maintenance treatment modeled as a time-varying covariate.

#### Exploratory MAIC-based indirect comparison

Individual patient-level data (IPD) from the serplulimab and placebo arms of the ASTRUM-005 trial were reconstructed using the Engauge Digitizer software to extract updated Kaplan-Meier survival curves from the 2024 ASCO Annual Meeting [3], followed by application of the IPDfromKM Shiny tool [8]. Baseline aggregate-level characteristics for the ASTRUM-005 population were obtained from the published article [5]. The MAIC was conducted following ISPOR best practices. Data of RCT-regimen cohort (derived from ASTRUM-005R) were reweighted to match the pivotal ASTRUM-005 trial baseline profile, and the effective sample size (ESS) was reported to reflect weighting’s impact on statistical power. Weighted Kaplan-Meier curves were generated to estimate adjusted OS.

## Results

**Fig. 1:**
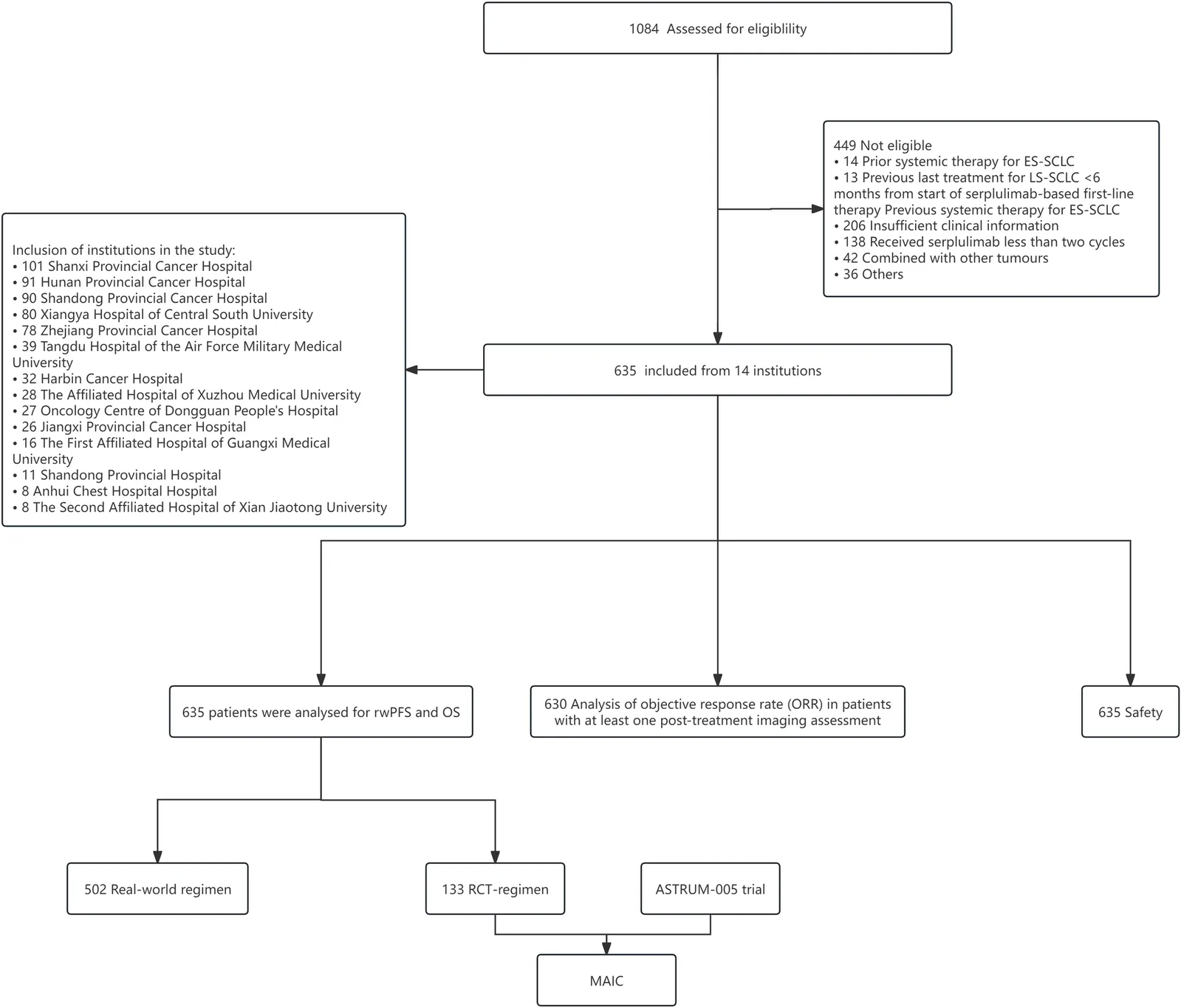
Study flowchart

We screened 1,084 patients from 14 tertiary academic medical sites and included 635 patients in the final analysis (Fig. 1). As of the cutoff data (December 31, 2024), the median follow-up duration was 14.90 months (range, 1.90-32.17 months).

### Baseline and treatment characteristics

Baseline characteristics are summarized in Table 1. The median age was 63 years (range, 30–84), with 277 (43.62%) aged ≥ 65 years. Most patients were male (*n* = 543, 85.51%) and smokers (*n* = 441, 69.45%). Notably, 75 patients (11.81%) had an ECOG PS ≥ 2, and 34.65% and 22.99% had liver and brain metastases, respectively, slightly higher than in the ASTRUM-005 trial. Based on treatment regimens, 133 patients (20.94%) were in the RCT-regimen cohort, and 502 patients (79.06%) were in the real-world regimen cohort. Baseline characteristics were generally balanced between the two cohorts.

**Table 1:** Baseline characteristics of included patients

Characteristics	All patients (*N* = 635)	RCT-regimen cohort (*N* = 133)	Real-world regimen cohort (*N* = 502)	*P* value
Age				
Years, median (range)	63 (30–84)	62 (48–82)	63 (30–84)	0.731
< 65 years, n (%)	358 (56.38)	74 (55.64)	284 (56.57)	0.847
≥ 65 years, n (%)	277 (43.62)	59 (44.36)	218 (43.43)	
Gender, n (%)				0.449
Male	543 (85.51)	111 (83.46)	432 (86.06)	
Female	92 (14.49)	22 (16.54)	70 (13.94)	
ECOG PS, n (%)				< 0.001*
0	167 (26.30)	37 (27.82)	130 (25.90)	
1	393 (61.89)	96 (72.18)	297 (59.16)	
2+	75 (11.81)	0 (0)^	75 (14.94)	
Smoking, n (%)				0.106
No	194 (30.55)	33 (24.81)	161 (32.07)	
Yes	441 (69.45)	100 (75.19)	341 (67.93)	
Former smoker	260 (40.94)	80 (60.15)	180 (35.86)	
Current smoker	177 (27.87)	19 (14.29)	158 (31.47)	
Unknown	4 (0.63)	1 (0.75)	3 (0.60)	
AJCC stage at diagnosis, n (%)				0.274
Stage III	53 (8.35)	8 (6.02)	45 (8.96)	
Stage IV	582 (91.65)	125 (93.98)	457 (91.04)	
Brain metastasis, n (%)				0.922
Yes	146 (22.99)	31 (23.31)	115 (22.91)	
No	489 (77.01)	102 (76.69)	387 (77.09)	
Liver metastasis, n (%)				0.403
Yes	220 (34.65)	42 (31.58)	178 (35.46)	
No	415 (65.35)	91 (68.42)	324 (64.54)	
Bone metastasis, n (%)				0.079
Yes	177 (27.87)	29 (21.80)	148 (29.48)	
No	458 (72.13)	104 (78.20)	354 (70.52)	

*P* values were calculated to compare baseline characteristics between the RCT-regimen cohort and the real-world regimen cohort

* Fisher’s exact test was used; ^ Refer to the key inclusion criteria from the ASTRUM-005 trial, no patients with ECOG PS ≥ 2 were included in the RCT-regimen cohort

ECOG PS, Eastern Cooperative Oncology Group performance status; AJCC, American Joint Committee on Cancer; RCT, randomized controlled trial

Regarding chemotherapy, 547 patients (86.14%) received etoposide plus carboplatin or cisplatin, with carboplatin more commonly used (*n* = 334, 52.60%) (Table 2). An additional 83 patients (13.07%) received other platinum agents with etoposide, such as lobaplatin or nedaplatin. Most patients (*n* = 548, 86.30%) completed at least four cycles of serplulimab-based combination therapy. Among 462 patients who completed at least four cycles without progression, 244 (52.81%) proceeded to receive maintenance therapy with serplulimab monotherapy, which may reflect relatively low compliance in real-world settings.

**Table 2:** Treatment characteristics of included patients

Characteristics	All patients (*N* = 635)	RCT-regimen cohort (*N* = 133)	Real-world regimen cohort (*N* = 502)
Chemotherapy regimen, n (%)			
Etoposide + platinum	630 (99.21)	133 (100)	497 (99.00)
Platinum agent			
Carboplatin	334 (52.60)	133 (100)	201 (40.04)
Cisplatin	213 (33.54)	0 (0)	213 (42.43)
Other platinum	83 (13.07)	0 (0)	83 (16.53)
Other chemotherapy regimen	5 (0.79)	0 (0)	5 (1.00)
Combination induction treatment sequence, n (%)			
Concurrent immunotherapy and chemotherapy	538 (84.72)	133 (100)	405 (80.68)
Sequential chemotherapy followed by immunotherapy	97 (15.28)	0 (0)*	97 (19.32)
Combination induction treatment cycles			
Median (range)	5 (2–6)	4 (2–4)	6 (2–6)
≤ 4 cycles, n (%)	291 (45.83)	133 (100)	158 (31.47)
> 4 cycles, n (%)	344 (54.17)	0 (0)^	344 (68.53)
Serplulimab maintenance treatment, n (%)			
Yes	264 (41.57)	48 (36.09)	216 (43.03)
No	371 (58.43)	85 (63.91)	286 (56.97)
Radiotherapy, n (%)			
Yes	201 (31.65)	36 (27.07)	165 (32.87)
Thoracic radiotherapy	124 (19.53)	24 (18.05)	100 (19.92)
Brain radiotherapy	78 (12.28)	13 (9.77)	65 (12.95)
Bone radiotherapy	25 (3.94)	3 (2.26)	22 (4.38)
Others	11 (1.73)	1 (0.75)	10 (1.99)
No	434 (68.35)	97 (72.93)	337 (67.13)

* According to the ASTRUM-005 trial treatment protocol, patients receiving sequential chemotherapy followed by immunotherapy were not included in the RCT-regimen cohort; ^ According to the ASTRUM-005 trial treatment protocol, patients receiving more than four cycles of combination induction treatment were not included in the RCT-regimen cohort

RCT, randomized controlled trial

### Real-world efficacy of serplulimab

#### OS

**Fig. 2:**
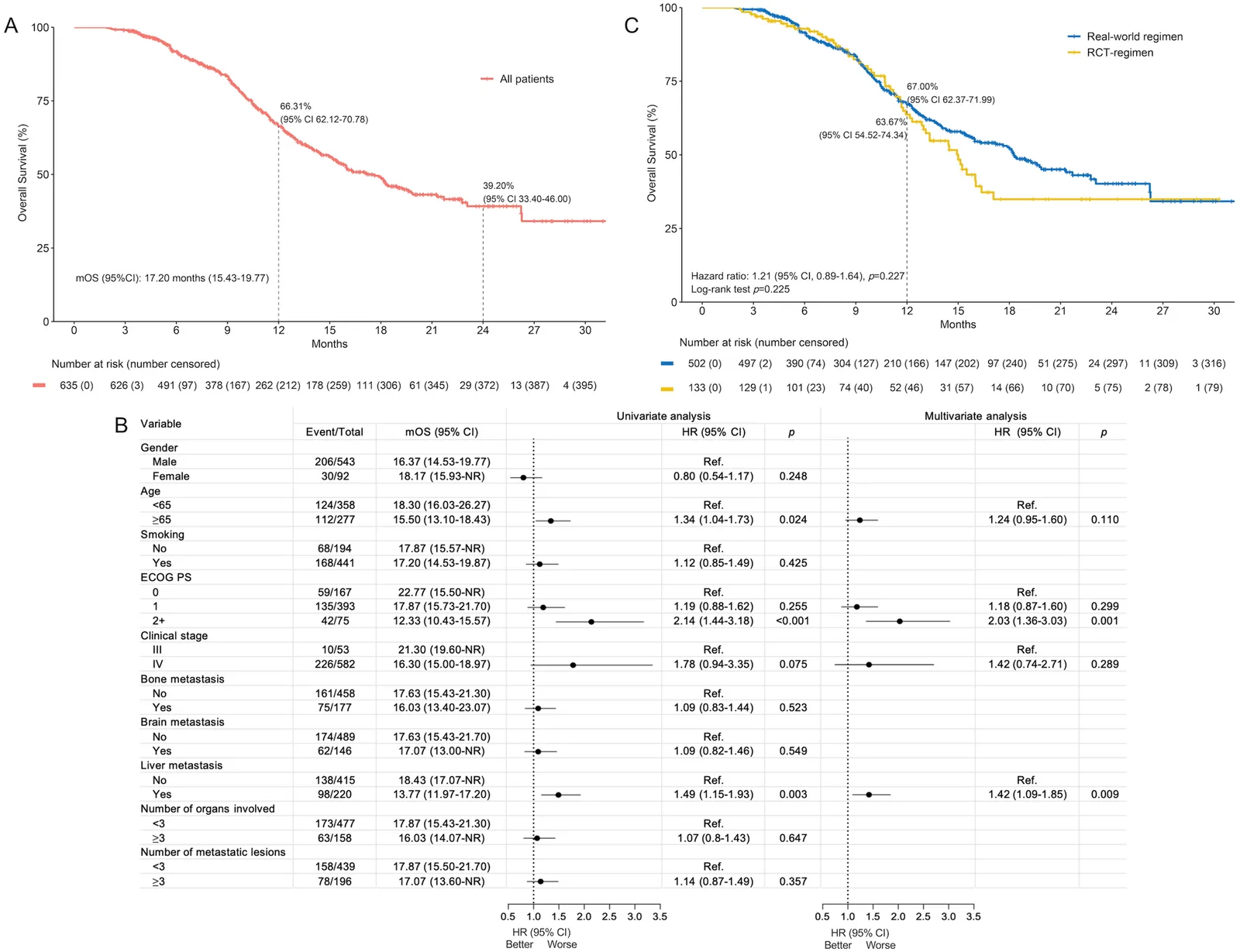
Overall survival (OS) outcomes in patients enrolled in the ASTRUM-005R study. (**A**) Kaplan-Meier survival curve for all patients. (**B**) Univariable and multivariable Cox proportional hazards models identifying independent risk factors associated with OS. (**C**) Kaplan-Meier survival comparison between patients treated with the RCT-regimen (defined per ASTRUM-005 protocol) and those receiving real-world regimens. RCT, randomized controlled trial; ECOG PS, Eastern Cooperative Oncology Group performance status; mOS, median overall survival; HR, hazard ratio; CI, confidence interval; Ref, reference

At cutoff, 236 deaths (37.17%) had occurred. The median OS for the entire cohort was 17.20 months (95% CI, 15.43–19.77), with a 1-year OS rate of 66.31% and a 2-year OS rate of 39.20% (Fig. 2A). On multivariable analysis, ECOG PS ≥ 2 and liver metastases independently predicted higher mortality (Fig. 2B). Median OS was 15.00 months in the RCT-regimen cohort and 18.27 months in the real-world cohort (log-rank *P* = 0.225; Fig. 2C). After excluding patients with ECOG PS ≥ 2, median OS in the real-world cohort increased to 19.87 months, numerically higher than that of the RCT-regimen cohort, but the difference remained statistically non-significant (log-rank *P* = 0.050; Additional file 2: Table S1, Additional file 3: Figure S1).

To address potential selection bias related to cohort definition, we supplemented a landmark analysis with the 4-cycle treatment completion time as the landmark, which was performed for all patients and separately for the subgroup excluding patients with ECOG PS ≥ 2. Consistent with the results prior to the landmark analysis, there was no significant difference in OS between the two cohorts both in the overall patient population and in the subgroup excluding those with ECOG PS ≥ 2 (Additional file 3: Figure S2).

#### rwPFS

**Fig. 3:**
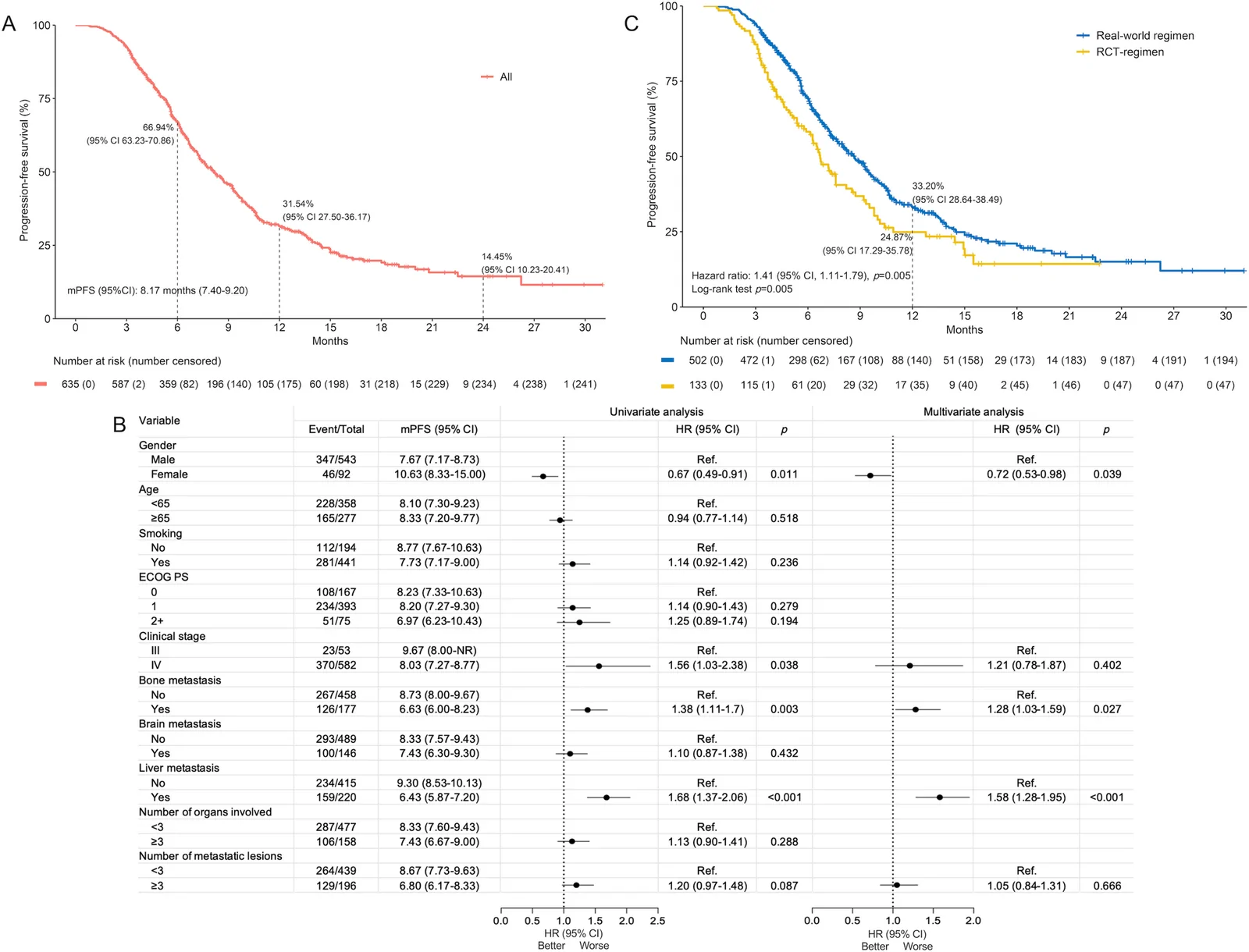
Real-world progression-free survival (rwPFS) outcomes of all included patients. (**A**) Kaplan-Meier survival curve for all patients. (**B**) Univariable and multivariable Cox proportional hazards models identifying independent risk factors associated with PFS. (C) Kaplan-Meier survival comparison between patients treated with the RCT-regimen (defined per ASTRUM-005 protocol) and those receiving real-world regimens. RCT, randomized controlled trial; ECOG PS, Eastern Cooperative Oncology Group performance status; mOS, median overall survival; HR, hazard ratio; CI, confidence interval; Ref, reference

By cutoff, 393 patients (61.89%) had experienced progression or death. Among 325 documented progressions, 95.38% were radiographically confirmed and 4.62% were based on clinical deterioration. The median rwPFS was 8.17 months (95% CI, 7.40–9.20), and 1-year rwPFS rate was 31.54% (Fig. 3A). Female sex predicted longer rwPFS, whereas baseline bone and liver metastases predicted shorter rwPFS (Fig. 3B). Median rwPFS was 6.70 months in the RCT-regimen cohort and 8.67 months in the real-world cohort (log-rank *P* = 0.005; Fig. 3C), the difference persisted after excluding ECOG PS ≥ 2 patients (log-rank *P* = 0.002; Additional file 2: Table S1, Additional file 3: Figure S3).

For rwPFS, the landmark analysis showed that there was a significant difference between the two cohorts both in all patients and in the subgroup excluding those with ECOG PS ≥ 2 before the landmark, and this significant difference persisted after the landmark, which was also consistent with the rwPFS analysis prior to the landmark analysis (Additional file 3: Figure S4).

#### Tumor response

Among 630 evaluable patients, ORR was 69.05% (435/630; 95% CI, 65.28–72.64) and disease control rate (DCR) was 95.40% (601/630; 95% CI, 93.46–96.90) (Table 3). ORR did not differ between cohorts: 72.52% in the RCT-regimen cohort and 68.14% in the real-world cohort (*P* = 0.334).

**Table 3:** Tumor response of patients with measurable baseline lesions

Tumor response	All patients (*N* = 630)	RCT-regimen cohort (*N* = 131)	Real-world regimen cohort (*N* = 499)	*P* value
Best overall response, n (%)				
CR	7 (1.11)	0 (0)	7 (1.40)	
PR	428 (67.94)	95 (72.52)	333 (66.73)	
SD	166 (26.35)	26 (19.85)	140 (28.06)	
PD	28 (4.44)	10 (7.63)	18 (3.61)	
NE	1 (0.16)	0 (0)	1 (0.20)	
ORR (95% CI)	69.05% (65.28–72.64)	72.52% (64.04–79.95)	68.14% (63.85–72.21)	0.334
DCR (95% CI)	95.40% (93.46–96.90)	92.37% (86.41–96.28)	96.19% (94.12–97.69)	0.063

A total of 630 patients with measurable baseline lesions were evaluable for tumor response. The *P* values compare the ORR and DCR between the RCT-regimen cohort and the real-world regimen cohort

CR, complete response; PR, partial response; SD, stable disease; PD, progressive disease; NE, not evaluable; ORR, objective response rate; DCR, disease control rate; CI, confidence interval; RCT, randomized controlled trial

### Safety profile

Overall, 341 patients (53.70%) experienced ≥ 1 AE; 304 (47.87%) had grade 1–2 and 103 (16.22%) reported grade ≥ 3 AEs (Table 4). The most frequent AEs were decreased white cell count (19.21%), alopecia (17.17%), and neutropenia (11.65%). The most common grade ≥ 3 AEs were decreased white cell count (5.51%), neutropenia (4.88%), and platelets decreased (2.20%). Treatment interruptions occurred in 90 patients (14.17%), and three AE-related deaths (0.47%) were attributed to infectious pneumonia, immune-related interstitial pneumonitis, and hepatic failure. Definite immune-related AEs (Definite-irAEs) occurred in 109 patients (17.17%), including 20 (3.15%) grade ≥ 3 Definite-irAEs; the most common Definite-irAEs were elevated alanine aminotransferase (ALT) (2.83%), thyroid function decreased (2.52%), and anemia (2.36%). Potential immune-related AEs (Potential-irAEs) occurred in 75 patients (11.81%), including 16 (2.52%) grade ≥ 3 Potential-irAEs; the most common Potential-irAEs were decreased white cell count (3.31%), nausea (2.36%), and neutropenia (2.05%). All AEs were managed per institutional protocols, and no new safety signals were observed (Additional file 2: Tables S2-3). Overall tolerability was acceptable.

**Table 4:** Summary of adverse events and stratified immune-related adverse events (Definite vs. Potential)

	Total	Grade 1–2	Grade ≥ 3
AEs	341 (53.70)	304 (47.87)	103 (16.22)
Incidence ≥ 5% AEs			
Decreased white cell count	122 (19.21)	87 (13.70)	35 (5.51)
Alopecia	109 (17.17)	109 (17.17)	0 (0)
Neutropenia	74 (11.65)	43 (6.77)	31 (4.88)
Anemia	65 (10.24)	58 (9.13)	7 (1.10)
Nausea	43 (6.77)	41 (6.46)	2 (0.31)
Platelets decreased	33 (5.20)	19 (2.99)	14 (2.20)
Definite-irAEs	109 (11.17)	93 (14.65)	20 (3.15)
Incidence ≥ 2% Definite-irAEs			
Elevated ALT	18 (2.83)	15 (2.36)	3 (0.47)
Thyroid function decreased	16 (2.52)	16 (2.52)	0 (0)
Anemia	15 (2.36)	11 (1.73)	4 (0.63)
Elevated AST	13 (2.05)	12 (1.89)	1 (0.16)
Potential-irAEs	75 (11.81)	60 (9.45)	16 (2.52)
Incidence ≥ 2% Potential -irAEs			
Decreased white cell count	21 (3.31)	18 (2.83)	3 (0.47)
Nausea	15 (2.36)	15 (2.36)	0 (0)
Neutropenia	13 (2.05)	4 (0.63)	9 (1.42)

Potential-irAEs are defined as events that cannot fully rule out immune-mediated synergistic or superimposed toxicities based on integrated medical records and detailed clinical timelines, whereas reactions without such possibilities are categorized as Definite-irAEs

AEs, adverse events; irAEs, immune-related adverse events; ALT, alanine aminotransferase; AST, aspartate aminotransferase

### Exploratory analyses

#### MAIC of OS versus ASTRUM-005 trial

**Fig. 4:**
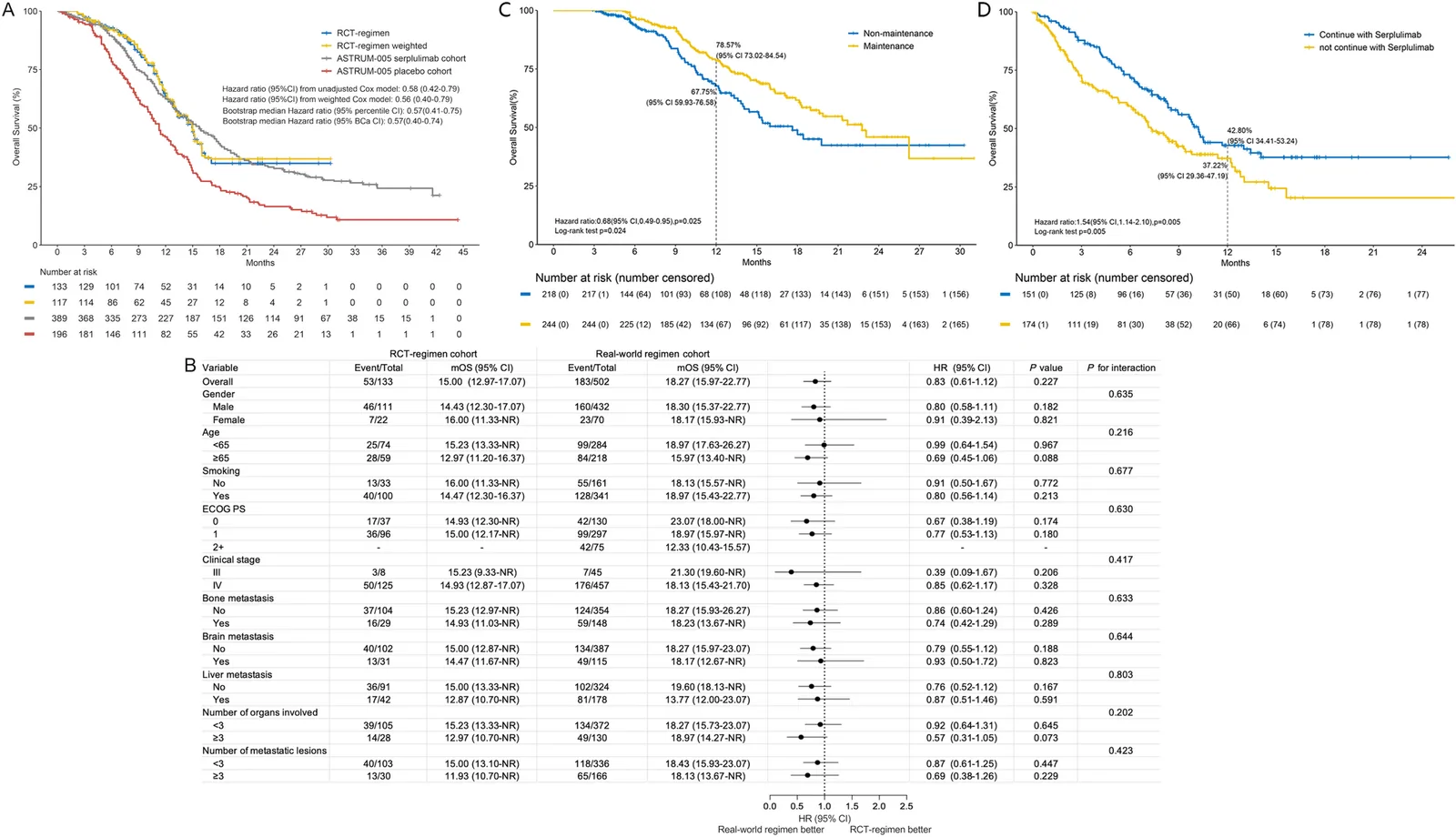
Exploratory analyses of survival outcomes. (**A**) MAIC-based external validation comparison of overall survival (OS) between the RCT-regimen (defined per ASTRUM-005 protocol) cohort and the ASTRUM-005 trial. Effective sample size refers to the number of independent unweighted individuals required to achieve the same precision as the weighted sample estimate. Note that in Kaplan-Meier analyses, the calculation of effective sample size differs to more accurately estimate the number at risk after weighting; therefore, the numbers at risk shown in the Kaplan-Meier plots differ from the effective sample size values reported in the text. (**B**) Subgroup analyses evaluating the heterogeneity of OS benefit across baseline variables between patients treated with the RCT-regimen and those receiving real-world regimens. (**C**) Impact of serplulimab maintenance therapy on OS among patients who completed induction treatment without progression. (**D**) OS analysis stratified by whether patients continued serplulimab-based therapy beyond first progression. MAIC, matching-adjusted indirect comparison; mOS, median overall survival; RCT, randomized controlled trial; HR, hazard ratio; CI, confidence interval; ECOG PS, Eastern Cooperative Oncology Group performance status; NR, not reached

To assess external validity, we conducted an MAIC between this RCT-regimen cohort and the ASTRUM-005 trial. As only aggregate-level baseline data from the ASTRUM‑005 trial were publicly available, with no IPD reported, the MAIC analysis was restricted to adjustment for shared and fully accessible variables between the two cohorts. Accordingly, the prespecified matching covariates comprised age, sex, smoking status, ECOG PS, and liver/brain metastases. After weighting, the ESS was 108 (-18.80%; Additional file 2: Table S4). There was no significant difference in OS between this RCT-regimen cohort with the ASTRUM-005 serplulimab arm (log-rank *P*_*unweighted*_ = 0.909; log-rank *P*_*weighted*_ = 0.784), but was superior to the ASTRUM-005 placebo arm (log-rank *P*_*unweighted*_ = 0.001; log-rank *P*_*weighted*_ <0.001), with a weighted HR of 0.56 (Fig. 4A).

#### Subgroup analysis of OS by regimen

Exploratory subgroup analysis across key baseline variables showed no difference in OS between the RCT-regimen and real-world regimens (HR = 1.21, 95% CI, 0.89–1.64). The magnitude of benefit with first-line serplulimab-based chemoimmunotherapy was consistent across all subgroups irrespective of regimen choice (Fig. 4B).

#### Impact of serplulimab maintenance therapy on OS

Both the ASTRUM-005 trial and other pivotal RCTs mandated up to two years of anti-PD-1/PD-L1 maintenance after 4–6 cycles of combination therapy, whereas real-world uptake is often limited by factors such as financial burden, cross-regional care barriers, or patient preference. Among 462 patients who completed ≥ 4 cycles without progression, serplulimab maintenance (*n* = 244) was associated with longer OS than no maintenance (22.77 months vs. 17.63 months; log-rank *P* = 0.024; Fig. 4C). At data cutoff, 100/244 patients (40.98%) in the maintenance group remained on therapy.

To further elucidate the true effect of serplulimab maintenance on OS and eliminate the confounding influence of guarantee-time bias on the results, we performed a time‑dependent Cox regression analysis. After adjustment with this model, serplulimab maintenance remained associated with a significant OS benefit (HR = 0.67, 95% CI: 0.47–0.83, *P* < 0.01).

#### Continuation of serplulimab beyond progression

Among 325 patients with documented progression, 151 (46.46%) continued serplulimab-based therapy. Post-progression survival (OS2) was longer with continuation versus non-continuation (median 10.20 vs. 7.30 months; log-rank *P* = 0.005), supporting a potential role for continued immunotherapy (Fig. 4D).

## Discussion

SCLC is aggressive, rapid growth, and prone to early metastasis. Landmark phase 3 trials (IMpower133 and CASPIAN) established anti-PD-L1 antibodies (atezolizumab or durvalumab) plus platinum-etoposide followed by immune maintenance as a new first-line standard for ES-SCLC [1]. However, the equivocal result of KEYNOTE-604 left the role of PD-1 inhibition uncertain [9]. The global phase 3 ASTRUM-005 trial brought the PD-1 inhibitor serplulimab into focus, demonstrating clinically meaningful efficacy with acceptable safety [5]. In this nationwide observational ASTRUM-005R study across 14 centers in China, we describe the real-world performance of first-line serplulimab plus chemotherapy. Among 635 patients, median OS was 17.20 months (95% CI, 15.43–19.77), median rwPFS was 8.17 months (95% CI, 7.40–9.20), and the ORR was 69.05%, with a manageable safety profile. An exploratory MAIC aligned outcomes from an RCT-concordant subset with ASTRUM-005, reinforcing the external validity of trial benefits under routine care. To our knowledge, this is the first large-scale real-world studies to validate the clinical outcomes of serplulimab outside the controlled setting of RCTs.

Consistent with prior studies, we found ECOG ≥ 2 to be independent predictor of higher mortality [10–12]. In our previously reported subgroup analysis, patients with ECOG PS ≥ 2 who received serplulimab plus chemotherapy achieved a median OS of 12.33 months [13], which was superior to historical chemotherapy-alone controls [14], suggesting that carefully selected patients with poor PS may still derive meaningful benefit under close monitoring. Patients with liver metastases exhibit particularly poor prognosis [15, 16], attributed to impaired hepatic function, enhanced tumor invasiveness, compromised treatment tolerance, and an immunosuppressive hepatic microenvironment that blunts the efficacy of immune checkpoint inhibitors [17, 18]. In contrast, bone metastasis mainly triggers local progression with manageable complications. In our cohort, liver metastases were an independent predictor of inferior OS and shorter rwPFS. Although outcomes were inferior to those without liver metastases, patients with liver involvement achieved a median OS of 13.77 months and a median rwPFS of 6.43 months, supporting the potential benefit of immunotherapy in this high-risk population. Bone metastasis was significantly associated with shortened rwPFS (*P* = 0.027), yet showed no significant correlation with inferior OS (*P* = 0.523). Mechanistically, bone metastases frequently precipitate early disease progression and clinical symptom deterioration, thereby directly reducing rwPFS [19–21]. In real-world clinical practice, local radiotherapy, symptomatic supportive care and sustained immunotherapy, combined with bone-modifying agents, targeted therapy and minimally invasive interventions, jointly facilitate durable local and systemic disease control. Combined with the long-tail survival effect of immunotherapy, patients may still achieve long-term survival benefits despite transient progression. Statistically, 126 of 177 patients reached the rwPFS endpoint with sufficiently mature data, whereas only 75 OS events occurred, resulting in inadequate data maturity and limited follow-up, which accounts for the nonsignificant association between bone metastasis and OS. Most studies suggest that female patients derive greater benefit from therapy compared to males, potentially due to lower smoking rates and hormonal factors [22–24]. Our analysis showed that female sex independently predicted longer rwPFS, whereas its association with OS was not significant (*P* = 0.248). In summary, these findings reinforce the biological heterogeneity of ES-SCLC and highlight the need for biomarker-driven, multi-omics studies to refine risk stratification and personalize therapy [25–27].

Maintenance therapy in SCLC is intended to suppress residual disease and delay progression. In our cohort of patients who completed ≥ 4 cycles of serplulimab-based chemoimmunotherapy without progression, serplulimab monotherapy maintenance was associated with longer OS versus no maintenance (median 22.77 vs. 17.63 months; log-rank *P* = 0.024) and a higher 12-month OS rate (78.57%, an absolute + 10.82%). Time-dependent Cox regression analysis further confirmed the authentic clinical benefit of serplulimab maintenance therapy. These findings align with reports of single-agent immune checkpoint inhibitor maintenance after first-line chemoimmunotherapy [1, 28, 29]. Exploratory analysis revealed that the serplulimab-continuation treatment beyond initial progression achieved significantly longer OS2 compared with the non-continuation group (log-rank *P* = 0.005). Sustained treatment may reactivate memory T cells and preserve residual antitumor immunity, with possible antigen spread to secondary tumor-associated targets [30, 31]; rational combinations could also help overcome emergent resistance [30, 32–35].

Additionally, our results showed no statistically significant difference in OS between patients treated with the trial-defined ASTRUM-005 regimen and those receiving investigator-determined real-world regimens, but the sensitivity analysis excluding patients with ECOG PS ≥ 2 revealed a numerically longer OS in the real-world cohort. This finding reinforces the established prognostic role of baseline performance status, while also raising the possibility that differences in treatment exposure may contribute to the observed numerical disparity. Specifically, the ASTRUM-005 protocol limited combination therapy (serplulimab plus carboplatin and etoposide) to four cycles. However, in real-world clinical practice, physicians often extend the duration of combination treatment up to six cycles based on patient tolerance and supportive evidence [36–38]; in our analysis, such patients were classified in the real-world cohort. Together with the MAIC showing concordant outcomes between our RCT-regimen subgroup and the pivotal trial, these findings support the external validity of the serplulimab-based regimen and indicate that serplulimab can deliver clinically meaningful survival benefits across complex real-world care settings.

The median rwPFS observed in this study was 8.17 months, which is numerically longer than the PFS reported in the ASTRUM-005 trial. Besides, the median rwPFS of the real-world cohort was longer than that of the RCT-regimen cohort. This phenomenon is not rare in the real world and may reflect several confounding factors. First, follow-up assessments in routine clinical practice are generally less standardized than in clinical trials; tumor progression may be detected later due to irregular imaging schedules or reliance on symptom-driven visits rather than protocol-mandated assessments. Second, real-world practice allows more flexible strategies: physicians may extend chemotherapy cycles, adjust agents based on treatment tolerance, and administer local radiotherapy. For instance, 18.05% of patients in the RCT‑regimen cohort received thoracic radiotherapy, whereas the ASTRUM-005 trial only permitted palliative radiotherapy for non-target lesions. This treatment difference may have conferred a modest favorable impact on survival outcomes in the RCT‑regimen cohort. Meanwhile, non-adherence to strict protocols together with supportive or complementary therapies may contribute to perceived disease stability and treatment persistence. Third, prolonged exposure to serplulimab in the real-world cohort may further enhance short-term clinical benefit. Furthermore, all patients included in this study received at least two cycles of serpulimab-based chemoimmunotherapy. This inclusion criterion ensured adequate drug exposure in patients but also excluded patients with early disease progression, which may explain why rwPFS was longer in this study than in the ASTRUM-005 trial.

AEs’ collection in real-world studies was inherently constrained by reliance on patient self-reporting and physician recognition, particularly for mild or transient events, potentially resulting in underestimation of AE incidence compared to RCTs [39, 40]. The spectrum of common AEs remained consistent with the pivotal ASTRUM-005 trial, encompassing decreased white cell count, alopecia, and neutrophils reduced^2^, with no novel safety signals identified. Furthermore, toxicity attribution for multimodal therapeutic strategies poses great challenges in routine real-world clinical practice. For irAEs, relying on complete medical records, we evaluated the temporal link between treatment interventions and AEs according to the clinical timeline of individual patients. Events such as decreased white blood cell count were classified as Potential-irAEs if immune synergistic mechanisms could not be excluded, and defined as Definite-irAEs otherwise. Notably, AE-related deaths occurred in both ASTRUM-005 and this real-world study, emphasizing the critical need for enhanced surveillance of toxicities during serplulimab treatment. Most AEs proved manageable, and serplulimab-based chemoimmunotherapy demonstrated favorable overall tolerability in this real-world population. However, the inherent limitations of AE reporting in routine clinical practice must be acknowledged when interpreting these safety findings.

This study has several limitations. First, this non-randomized real-world study was conducted exclusively in Chinese centers, making it subject to selection bias and unmeasured confounding, and limiting its generalizability to non-Chinese populations. Second, although we categorized patients into RCT-regimen and real-world regimen cohorts based on the ASTRUM-005 protocol, patients receiving > 4 cycles who have better performance status by definition were included in the real-world cohort. This differential allocation introduces immortal time bias and treatment-intensity bias, as prolonged drug exposure in fitter patients could have contributed to the numerically improved outcomes observed in the real-world group. Third, given that only aggregated baseline data from the ASTRUM-005 trial were available, MAIC adjustment was limited to six core covariates fully documented in both our study and the ASTRUM-005 trial. Although these are established prognostic factors for ES-SCLC, residual confounding from unmeasured variables cannot be excluded, potentially biasing the OS comparison. This limitation is inherent to indirect comparisons using published trial data. Fourth, this was a large-sample real-world study across 14 clinical centers. Owing to inherent differences in medical workflows and resource allocation, imaging assessments were individualized and could not be standardized uniformly across all centers. Accordingly, sensitivity analyses based on standardized assessment intervals were not feasible, which may have delayed progression detection and led to overestimation of PFS duration. Despite these limitations, this multicenter real-world study provides valuable complementary evidence to support the clinical effectiveness and feasibility of serplulimab-based first-line therapy in ES-SCLC across diverse treatment scenarios.

## Conclusions

In conclusion, this large-scale, multicenter, observational study provides compelling real-world evidence confirming the effectiveness and tolerability of serplulimab-based chemoimmunotherapy as a first-line treatment for ES-SCLC. Across a heterogeneous patient population and varying clinical practices, serplulimab demonstrated consistent survival benefits, with a median OS of 17.20 months and an ORR of 69.05%, with manageable safety profiles consistent with prior trial data. These findings reinforce the pivotal results of the ASTRUM-005 trial. Additionally, the comparable outcomes observed between the RCT-regimen and real-world cohorts, along with consistent results from sensitivity and subgroup analyses, highlight the feasibility of extending this treatment strategy beyond rigid trial settings. The MAIC analysis further validated the real-world effectiveness against the ASTRUM-005 benchmark, offering a robust methodological bridge between randomized and observational evidence. Additionally, exploratory findings suggest that serplulimab maintenance or continuation beyond initial progression may yield incremental survival benefits and warrant further prospective validation. Collectively, this study represents a critical step toward evidence-based immunotherapy for ES-SCLC in the real world.

## Supplementary Information

Below is the link to the electronic supplementary material.

Supplementary Material 1: Additional File 1: STROBE Checklist

Supplementary Material 2: Additional File 2: Table S1-S4.

Supplementary Material 3: Additional File 3: Figures S1-S4

## Data Availability

All de-identified datasets used for the analyses presented in this study, processed in full compliance with patient privacy protection regulations and institutional ethical requirements, are deposited in the Zenodo repository and are available only upon reasonable request from the corresponding author (Lin Wu), with the assigned permanent DOI (https://doi.org/10.5281/zenodo.19549314) [41].

## Abbreviations

ES-SCLC: Extensive-stage small cell lung cancer
MAIC: Matching-adjusted indirect comparison
OS: overall survival
rwPFS: real-world progression-free survival
ORR: Objective response rate
RCT: randomized controlled trial
mAb: monoclonal antibody
ECOG PS: Eastern Cooperative Oncology Group performance status
RWE: real-world evidence
RWD: real-world data
DCR: disease control rate
ALT: alanine aminotransferase
AST: aspartate aminotransferase
OS2: post-progression survival
ICI: immune checkpoint inhibitor
AE: Adverse event
EDC: electronic data capture
CR: complete response
PR: partial response
S.D.: standard deviation
IQRs: interquartile ranges
ORs: odds ratios
IPD: Individual patient-level data
ESS: effective sample size
