# Supplementary material for: Real-world serplulimab plus chemotherapy for extensive-stage small cell lung cancer in china: an external validation of ASTRUM-005 trial
Source: BMC Med. 2026 Jul 15;24:558. doi: 10.1186/s12916-026-05055-9 (PMC13651263; doi:10.1186/s12916-026-05055-9)
Supplement: Supplementary file 2 [file 12916_2026_5055_MOESM2_ESM.docx]

**Supplementary Tables**

**Table S1.** Baseline characteristics of patients included in the sensitivity analysis excluding those with ECOG PS ≥2

|  | RCT-regimen cohort (N=133) | Real-world regimen cohort (N=427) | *P* value |
| --- | --- | --- | --- |
| Age |  |  |  |
| Years, median (range) | 62 (48-82) | 62(30-84) | 0.263 |
| <65 years, n (%) | 74 (55.64) | 253 (59.25) | 0.461 |
| ≥65 years, n (%) | 59 (44.36) | 174 (40.75) |  |
| Gender, n (%) |  |  | 0.252 |
| Male | 111 (83.46) | 373 (87.35) |  |
| Female | 22 (16.54) | 54 (12.65) |  |
| ECOG PS, n (%) |  |  | 0.563 |
| 0 | 37 (27.82) | 130 (30.44) |  |
| 1 | 96 (72.18) | 297 (69.56) |  |
| Smoking, n (%) |  |  | 0.135 |
| No | 33 (24.81) | 135 (31.62) |  |
| Yes | 100 (75.19) | 292 (68.38) |  |
| Former smoker | 80 (60.15) | 140 (32.79) |  |
| Current smoker | 19 (14.29) | 150 (35.13) |  |
| Unknown | 1 (0.75) | 2 (0.47) |  |
| AJCC stage at diagnosis, n (%) |  |  | 0.137 |
| Stage III | 8 (6.02) | 44 (10.30) |  |
| Stage IV | 125 (93.98) | 383 (89.70) |  |
| Brain metastasis, n (%) |  |  | 0.545 |
| Yes | 31 (23.31) | 89 (20.84) |  |
| No | 102 (76.69) | 338 (79.16) |  |
| Liver metastasis, n(%) |  |  | 0.612 |
| Yes | 42 (31.58) | 145 (33.96) |  |
| No | 91 (68.42) | 282 (66.04) |  |
| Bone metastasis,n(%) |  |  | 0.102 |
| Yes | 29 (21.80) | 124 (29.04) |  |
| No | 104 (78.20) | 303 (70.96) |  |

*P* values were calculated to compare baseline characteristics between the RCT-regimen cohort and the real-world regimen cohort after excluding patients with ECOG PS ≥2.

ECOG PS, Eastern Cooperative Oncology Group performance status; AJCC, American Joint Committee on Cancer; RCT, randomized controlled trial.

**Table S2.** Adverse events of serplulimab-based chemoimmunotherapy for ES-SCLC patients in real-world practice settings

|  | Total | Grade 1-2 | Grade ≥3 |
| --- | --- | --- | --- |
| AEs | 341 (53.70) | 304 (47.87) | 103 (16.22) |
| Decreased white cell count | 122 (19.21) | 87 (13.70) | 35 (5.51) |
| Alopecia | 109 (17.17) | 109 (17.17) | 0 (0) |
| Neutropenia | 74 (11.65) | 43 (6.77) | 31 (4.88) |
| Anemia | 65 (10.24) | 58 (9.13) | 7 (1.10) |
| Nausea | 43 (6.77) | 41 (6.46) | 2 (0.31) |
| Platelets decreased | 33 (5.20) | 19 (2.99) | 14 (2.20) |
| Elevated ALT | 29 (4.57) | 26 (4.09) | 3 (0.47) |
| Elevated AST | 29 (4.57) | 24 (3.78) | 5 (0.79) |
| Adynamia | 26 (4.09) | 26 (4.09) | 0 (0) |
| Lymphocyte count decreased | 24 (3.78) | 24 (3.78) | 0 (0) |
| Thyroid function decreased | 19 (2.99) | 19 (2.99) | 0 (0) |
| Vomiting | 17 (2.68) | 15 (2.36) | 2 (0.31) |
| Cough | 16 (2.52) | 15 (2.36) | 1 (0.16) |
| Hyperglycaemia | 15 (2.36) | 13 (2.05) | 2 (0.31) |
| Bone marrow depression | 11 (1.73) | 3 (0.47) | 8 (1.26) |
| Pneumonia | 10 (1.57) | 6 (0.94) | 4 (0.63) |
| Decreased appetite | 10 (1.57) | 10 (1.57) | 0 (0) |
| Abdominal distension | 9 (1.42) | 9 (1.42) | 0 (0) |
| Creatinine increased | 9 (1.42) | 9 (1.42) | 0 (0) |
| Interstitial pneumonia | 6 (0.94) | 5 (0.79) | 1 (0.16) |
| Rash | 6 (0.94) | 6 (0.94) | 0 (0) |
| Pruritus | 6 (0.94) | 6 (0.94) | 0 (0) |
| Diarrhea | 5 (0.79) | 5 (0.79) | 0 (0) |
| Productive cough | 5 (0.79) | 4 (0.63) | 1 (0.16) |
| Blood lactate dehydrogenase increased | 5 (0.79) | 5 (0.79) | 0 (0) |
| Radiation pneumonitis | 4 (0.63) | 2 (0.31) | 2 (0.31) |
| Chest tightness | 4 (0.63) | 4 (0.63) | 0 (0) |
| Chest pain | 4 (0.63) | 4 (0.63) | 0 (0) |
| Hepatic function decreased | 3 (0.47) | 2 (0.31) | 1 (0.16) |
| Hepatitis | 3 (0.47) | 2 (0.31) | 1 (0.16) |
| Hypertriglyceridaemia | 3 (0.47) | 3 (0.47) | 0 (0) |
| Hoarseness | 3 (0.47) | 3 (0.47) | 0 (0) |
| Myocardial injury | 3 (0.47) | 2 (0.31) | 1 (0.16) |
| Constipation | 2 (0.31) | 2 (0.31) | 0 (0) |
| Singultus | 2 (0.31) | 2 (0.31) | 0 (0) |
| Hypercholesteremia | 2 (0.31) | 2 (0.31) | 0 (0) |
| Arthralgia | 2 (0.31) | 2 (0.31) | 0 (0) |
| Oral pain | 2 (0.31) | 2 (0.31) | 0 (0) |
| Hazy vision | 2 (0.31) | 2 (0.31) | 0 (0) |
| Headache | 2 (0.31) | 2 (0.31) | 0 (0) |
| Pain knee | 2 (0.31) | 2 (0.31) | 0 (0) |
| Pain pharynx | 2 (0.31) | 1 (0.16) | 1 (0.16) |
| Primary hypoadrenalism | 2 (0.31) | 2 (0.31) | 0 (0) |
| Back pain | 1 (0.16) | 1 (0.16) | 0 (0) |
| Hypopituitalism | 1 (0.16) | 1 (0.16) | 0 (0) |
| Hyponatraemia | 1 (0.16) | 1 (0.16) | 0 (0) |
| Amylase increased | 1 (0.16) | 1 (0.16) | 0 (0) |
| Fever | 1 (0.16) | 1 (0.16) | 0 (0) |
| Lung infection | 1 (0.16) | 1 (0.16) | 0 (0) |
| Triglyceride increased | 1 (0.16) | 0 (0) | 1 (0.16) |
| Hepatic insufficiency | 1 (0.16) | 1 (0.16) | 0 (0) |
| Haemoptysis | 1 (0.16) | 1 (0.16) | 0 (0) |
| Sudoresis | 1 (0.16) | 1 (0.16) | 0 (0) |
| Dyspnea | 1 (0.16) | 1 (0.16) | 0 (0) |
| Respiratory failure | 1 (0.16) | 0 (0) | 1 (0.16) |
| Myalgia | 1 (0.16) | 0 (0) | 1 (0.16) |
| Hyperthyroidism | 1 (0.16) | 0 (0) | 1 (0.16) |
| Shoulder blade pain | 1 (0.16) | 1 (0.16) | 0 (0) |
| Mental aberration | 1 (0.16) | 1 (0.16) | 0 (0) |
| Mouth ulceration | 1 (0.16) | 1 (0.16) | 0 (0) |
| Endocrine toxicity | 1 (0.16) | 1 (0.16) | 0 (0) |
| Disorder coagulation | 1 (0.16) | 1 (0.16) | 0 (0) |
| Polypnoea | 1 (0.16) | 1 (0.16) | 0 (0) |
| Hypoxic encephalopathy | 1 (0.16) | 0 (0) | 1 (0.16) |
| Neuralgia | 1 (0.16) | 1 (0.16) | 0 (0) |
| Kidney function abnormal | 1 (0.16) | 1 (0.16) | 0 (0) |
| Nephritis | 1 (0.16) | 0 (0) | 1 (0.16) |
| Infusion reaction | 1 (0.16) | 0 (0) | 1 (0.16) |
| Weight loss | 1 (0.16) | 1 (0.16) | 0 (0) |
| Stomachache | 1 (0.16) | 1 (0.16) | 0 (0) |
| Knee swelling | 1 (0.16) | 1 (0.16) | 0 (0) |
| Palpitation | 1 (0.16) | 1 (0.16) | 0 (0) |
| Thoracic back pain | 1 (0.16) | 0 (0) | 1 (0.16) |
| Chest discomfort | 1 (0.16) | 1 (0.16) | 0 (0) |
| Blood potassium decreased | 1 (0.16) | 1 (0.16) | 0 (0) |
| Elevated blood glucose | 1 (0.16) | 0 (0) | 1 (0.16) |
| Dry throat | 1 (0.16) | 1 (0.16) | 0 (0) |
| Fungal infection | 1 (0.16) | 1 (0.16) | 0 (0) |
| Sensory peripheral neuropathy | 1 (0.16) | 0 (0) | 1 (0.16) |
| Elevated total bilirubin | 1 (0.16) | 1 (0.16) | 0 (0) |

AEs, adverse events; ALT, alanine aminotransferase; AST, aspartate aminotransferase.

**Table S3.** Immune-related adverse events of serplulimab-based chemoimmunotherapy for ES-SCLC patients in real-world practice settings

|  | Total | Grade 1-2 | Grade ≥3 |
| --- | --- | --- | --- |
| irAEs | 160 (25.20) | 133 (20.94) | 34 (5.35) |
| Decreased white cell count | 29 (4.57) | 25 (3.94) | 4 (0.63) |
| Elevated ALT | 22 (3.46) | 19 (2.99) | 3 (0.47) |
| Elevated AST | 18 (2.83) | 16 (2.52) | 2 (0.31) |
| Thyroid function decreased | 16 (2.52) | 16 (2.52) | 0 (0) |
| Nausea | 15 (2.36) | 15 (2.36) | 0 (0) |
| Anemia | 15 (2.36) | 11 (1.73) | 4 (0.63) |
| Neutropenia | 15 (2.36) | 5 (0.79) | 10 (1.57) |
| Hyperglycaemia | 11 (1.73) | 9 (1.42) | 2 (0.31) |
| Adynamia | 8 (1.26) | 8 (1.26) | 0 (0) |
| Vomiting | 7 (1.10) | 7 (1.10) | 0 (0) |
| Platelets decreased | 7 (1.10) | 4 (0.63) | 3 (0.47) |
| Creatinine increased | 5 (0.79) | 5 (0.79) | 0 (0) |
| Pruritus | 5 (0.79) | 5 (0.79) | 0 (0) |
| Decreased appetite | 5 (0.79) | 5 (0.79) | 0 (0) |
| Interstitial pneumonia | 5 (0.79) | 4 (0.63) | 1 (0.16) |
| Abdominal distension | 3 (0.47) | 3 (0.47) | 0 (0) |
| Rash | 3 (0.47) | 3 (0.47) | 0 (0) |
| Pneumonia | 3 (0.47) | 1 (0.16) | 2 (0.31) |
| Singultus | 2 (0.31) | 2 (0.31) | 0 (0) |
| Hepatic function decreased | 2 (0.31) | 2 (0.31) | 0 (0) |
| Hypertriglyceridaemia | 2 (0.31) | 2 (0.31) | 0 (0) |
| Cough | 2 (0.31) | 2 (0.31) | 0 (0) |
| Primary hypoadrenalism | 2 (0.31) | 2 (0.31) | 0 (0) |
| Hepatitis | 2 (0.31) | 1 (0.16) | 1 (0.16) |
| Bone marrow depression | 2 (0.31) | 1 (0.16) | 1 (0.16) |
| Myocardial injury | 2 (0.31) | 1 (0.16) | 1 (0.16) |
| Radiation pneumonitis | 1 (0.16) | 0 (0) | 1 (0.16) |
| Myalgia | 1 (0.16) | 0 (0) | 1 (0.16) |
| Hyperthyroidism | 1 (0.16) | 0 (0) | 1 (0.16) |
| Nephritis | 1 (0.16) | 0 (0) | 1 (0.16) |
| Infusion reaction | 1 (0.16) | 0 (0) | 1 (0.16) |
| Sensory peripheral neuropathy | 1 (0.16) | 0 (0) | 1 (0.16) |
| Hypopituitalism | 1 (0.16) | 1 (0.16) | 0 (0) |
| Fever | 1 (0.16) | 1 (0.16) | 0 (0) |
| Diarrhea | 1 (0.16) | 1 (0.16) | 0 (0) |
| Hypercholesteremia | 1 (0.16) | 1 (0.16) | 0 (0) |
| Arthralgia | 1 (0.16) | 1 (0.16) | 0 (0) |
| Dyspnea | 1 (0.16) | 1 (0.16) | 0 (0) |
| Oral pain | 1 (0.16) | 1 (0.16) | 0 (0) |
| Hazy vision | 1 (0.16) | 1 (0.16) | 0 (0) |
| Knee swelling | 1 (0.16) | 1 (0.16) | 0 (0) |
| Palpitation | 1 (0.16) | 1 (0.16) | 0 (0) |
| Chest discomfort | 1 (0.16) | 1 (0.16) | 0 (0) |
| Fungal infection | 1 (0.16) | 1 (0.16) | 0 (0) |
| Elevated total bilirubin | 1 (0.16) | 1 (0.16) | 0 (0) |

irAEs, immune-related adverse events; ALT, alanine aminotransferase; AST, aspartate aminotransferase.

**Table S4.** Baseline characteristics of the RCT-regimen cohort before and after weighting in the MAIC analysis compared with the ASTRUM-005 trial

|  | RCT-regimen cohort | | ASTRUM-005 | |
| --- | --- | --- | --- | --- |
|  | Before Matching (N=133) | After Matching (N=107)* | Serplulimab (N=389) | Placebo (N=196) |
| Age |  |  |  |  |
| <65 years, % | 55.64 | 60.71 | 60.41 | 60.71 |
| ≥65 years, % | 44.36 | 39.29 | 39.59 | 39.29 |
| Gender, % |  |  |  |  |
| Male | 83.46 | 83.67 | 81.49 | 83.67 |
| Female | 16.54 | 16.33 | 18.51 | 16.33 |
| ECOG PS, % |  |  |  |  |
| 0 | 27.82 | 16.33 | 18.25 | 16.33 |
| 1 | 72.18 | 83.67 | 81.75 | 83.67 |
| Smoking, % |  |  |  |  |
| No | 24.81 | 17.86 | 20.82 | 17.86 |
| Yes | 75.19 | 82.14 | 79.18 | 82.14 |
| Brain metastasis, % |  |  |  |  |
| Yes | 23.31 | 14.29 | 12.85 | 14.29 |
| No | 76.69 | 85.71 | 87.15 | 85.71 |
| Liver metastasis, % |  |  |  |  |
| Yes | 31.58 | 26.02 | 25.45 | 26.02 |
| No | 68.42 | 73.98 | 74.55 | 73.98 |

Key baseline variables considered for matching included age, sex, smoking status, ECOG performance status, and the presence of liver or brain metastases.

* Effective sample size was computed as the square of the summed weights divided by the sum of the squared weights.

MAIC, matching-adjusted indirect comparison; ECOG PS, Eastern Cooperative Oncology Group performance status; RCT, randomized controlled trial.
