# Supplementary material for: Real-world serplulimab plus chemotherapy for extensive-stage small cell lung cancer in china: an external validation of ASTRUM-005 trial
Source: BMC Med. 2026 Jul 15;24:558. doi: 10.1186/s12916-026-05055-9 (PMC13651263; doi:10.1186/s12916-026-05055-9)
Supplement: Supplementary file 3 [file 12916_2026_5055_MOESM3_ESM.docx]

**Supplementary Figures**

**Figure S1. Sensitivity analysis of overall survival (OS) excluding patients with ECOG PS ≥2**

Kaplan-Meier survival curves comparing OS between the RCT-regimen cohort and the real-world regimen cohort after excluding patients with Eastern Cooperative Oncology Group performance status (ECOG PS) ≥2.

**Figure S2. Landmark analysis of OS outcomes.**

(A) Kaplan-Meier survival curves from the landmark analysis comparing OS between the RCT-regimen cohort and the real-world regimen cohort. (B) Kaplan-Meier survival curves from the landmark analysis comparing OS between the RCT-regimen cohort and the real-world regimen cohort after excluding patients with ECOG PS ≥2.

**Figure S3. Sensitivity analysis of real-world progression-free survival (rwPFS) excluding patients with ECOG PS ≥2**

Kaplan-Meier survival curves comparing rwPFS between the RCT-regimen cohort and the real-world regimen cohort after excluding patients with Eastern Cooperative Oncology Group performance status (ECOG PS) ≥2.

**Figure S4. Landmark analysis of rwPFS outcomes.**

(A) Kaplan-Meier survival curves from the landmark analysis comparing rwPFS between the RCT-regimen cohort and the real-world regimen cohort. (B) Kaplan-Meier survival curves from the landmark analysis comparing rwPFS between the RCT-regimen cohort and the real-world regimen cohort after excluding patients with ECOG PS ≥2.
